# Acute Kidney Injury in the Critically Ill Patient

**DOI:** 10.1155/2013/529524

**Published:** 2013-06-23

**Authors:** Manuel E. Herrera-Gutiérrez, Gemma Seller-Pérez, Javier Maynar-Moliner, José A. Sánchez-Izquierdo-Riera, Anibal Marinho, José Luis Do pico

**Affiliations:** ^1^Department of Critical Care Medicine, University Hospital Carlos Haya, Málaga, Spain; ^2^University of Malaga School of Medicine, Spain; ^3^Department of Critical Care Medicine, Santiago Hospital, Vitoria, Spain; ^4^Department of Critical Care Medicine, Hospital 12 de Octubre, Madrid, Spain; ^5^Department of Critical Care Medicine, Centro Hospitalario de Porto, Portugal; ^6^Department of Critical Care Medicine, Hospital Municipal de Necochea, Argentina

Epidemiology of acute kidney injury (AKI) is supposedly a well-characterized topic. Large population studies developed in the 1990s demonstrated a higher incidence of AKI than previously suspected. More importantly, this incidence was higher among critically ill patients while mortality rates rose dramatically in this setting. In these studies an overall incidence of around five per cent of AKI was found in ICU with an accompanying mortality in over fifty per cent of cases.

Recently we have witnessed a significant change in this field with the development of a classification system for staging kidney damage (RIFLE, AKIN, and KDIGO). These systems have marked a shift from static to a dynamic concept of AKI based in changes in kidney function regardless of the net functional capability found in our patients. This change has had an important impact on the way intensivists look at the kidney problem (focusing now on early secondary prevention in order to arrest the process), but this has created an unexpected problem due to a dramatic increase in the number of patients that we label as AKI. To clarify the real impact of AKI we must admit that all the figures derived from the early studies are no longer useful and consequently new epidemiological studies have been developed all over the world. 

In an interesting review “*Epidemiology of acute kidney injury in the intensive care unit*” by J. Case et al. published in a recent issue of *Clinical Care Research and Practice*, the authors highlighted the complexity of this problem that is yet to be solved. Following J. Case et al., incidence of AKI in critically ill patients has risen during the past decade due to increased acuity as well as increased recognition. Following recent standards, overall incidence ranges from 20% to 50% but, as this paper emphasizes, current rates are dependent on the specific ICU population under study, with lower rates for scheduled surgery and higher rates in septic patients.

A note of interest is that even when measured incidence of AKI varied from five per cent to fifty per cent, reported mortality was still around 50% and this makes us question the real impact of AKI detection. What is required in the near future is to fully understand AKI, define its incidence among specific population groups, and evaluate the real impact of this improved capability of AKI detection.

If we consider the aforementioned impact of new stage systems and the fact that we lack effective treatment to avoid or limit kidney damage, we are faced with the prospect of providing effective preventive measures. To be effective, this approach requires, primarily, the identification of high-risk populations, but until now, studies showed different risk factors dependent on the geographical setting, the population addressed, and the particular selection of the investigators. In regards to this problem, an interesting meta-analysis by Cartin-Ceba et al. “*Risk factors for development of Acute kidney injury in critically ill patients: A systematic review and meta-analysis of observational studies*” analyzed data extracted from 31 studies comprising 504,535 critically ill patients from a wide variety of ICUs. This meta-analysis showed a significantly increased risk of AKI in the following: older patients, those admitted after surgery, more severely ill, and patients with higher baseline creatinine or when nephrotoxic drugs are used.

Historically the screening and diagnosis of AKI have been based on a surrogate of glomerular filtration rate, serum creatinine. This is regarded as the most practical approach considering that creatinine is affordable and universally available and has been potentiated with the application of new staging systems based on changes of serum creatinine as markers for proportional changes in creatinine clearance. In this special issue, G. Seller-Pérez et al. expose the potential pitfalls of this approach in a review titled “*Estimating kidney function in the critically ill patients*”. 

When basal creatinine is not available in the RIFLE staging system, an estimation extracted from the MDRD equation is an acceptable alternative. The estimation of creatinine clearance by different equations is an accepted guide for the dosage of different drugs in the ICU setting, even when clinicians are well aware of the fact that these equations were developed for the use on patients with chronic kidney disease and have been scarcely validated for acutely ill patients. In regards to this issue, C. Kirwan et al. presented a study (*Estimated glomerular filtration rate correlates poorly with four-hour creatinine clearance in critically ill patients with acute kidney injury*) aiming to test whether creatinine or cystatin C based eGFR equations offer an accurate representation of creatinine clearance in critically ill patients with AKI and not surprisingly found that these equations were unreliable in this setting. The authors concluded (and we definitely agree with their position) that these equations should not be used to describe renal function in ICU patients with AKI and that new standards of validation for these equations are required.

If we consider the importance of early prevention and the drawbacks of creatinine as diagnostic tool for AKI (mainly the delay between damage and change of serum levels) it is easy to understand the recent interest in the study of new markers of kidney damage (both functional or structural damage biomarkers). The role of the most promising biomarkers [neutrophil gelatinase-associated lipocalin (NGAL), cystatin C (Cys C), kidney injury molecule-1 (KIM-1), Interleukin-18 (IL-18) and liver-type fatty acids binding protein (L-FABP)] is explored by Tsigou et al. in the interesting review paper “*Role of new biomarkers: functional and structural damage*.” This paper highlights important issues when evaluating the role of new biomarkers for AKI and some of these are also seen in the study that A. Royakkers et al. present in this issue (*Systemic and urinary neutrophil gelatinase-associated lipocalin are poor predictors of acute kidney injury in unselected critically Ill patients*). These authors did not find urinary or plasma NGAl useful as markers for AKI development in an unselected population, but (as they point out in their discussion) these results conflict with other published studies focusing on heterogeneous ICU populations. Biomarkers for AKI are still in a development stage and we need further studies in order to define their actual capability for early detection, possible interferences in their values, ideal cut-off values in different clinical settings and whether the simultaneous use of a battery of biomarkers, could have an impact on kidney injury detection.

New trends must be found not only in AKI definition or diagnosis but in other areas too. Until recently, aggressive fluid administration as a mean to ensure an adequate perfusion was the cornerstone of the intensivists' earlier intervention. This approach was considered the most critical aspect regarding AKI prevention. After the publication of some important studies demonstrating that in fact this approach may not be adequate (and can even be detrimental) we must now reassess the role of volume resuscitation in the critically ill patient and define which patients are candidates for an aggressive fluid resuscitation and which fluid should be used for each scenario. These questions and much more (evaluation of volume status in critically ill patients, disturbances in the distribution of body water or its effect on creatinine or urea kinetics, i.e.) are addressed in the review from M. Labib et al. titled “*Volume management in the critically ill patient with acute kidney injury*”.

Finally, A. Leung et al. in the original study titled “*A retrospective review of the use of regional citrate anticoagulation in continuous venovenous hemofiltration (CVVH) for critically ill patients*” address another current issue, the best anticoagulation strategy for keeping permeable renal replacement (RRT) circuits. Recently different investigators presented interesting results demonstrating that citrate anticoagulation is efficient and safe even in circumstances that were previously prohibited. The only important drawback for this strategy is the requirement of a serum ionic calcium control and the use for dialysis and reinfusion fluids with a different composition (devoid of calcium and with a lower concentration of bicarbonate) from the commercial solutions available. A. Leung presented a protocol devised for a widely available RRT monitor that is safe and easy to perform with excellent results in terms of circuit survival.

The papers on this topic present to the reader a comprehensive picture of the acute kidney injury problem in the ICU setting: dire necessity for early detection beside a lack of adequate tools for early diagnosis; promising biomarkers that raise expectation but are still in the developmental stage and far from being a clinical routine; the fact that almost every other patient will develop AKI, but we have no effective measures to prevent or treat it and, underlying all these facts, knowledge that AKI worsens dramatically the prognosis of our patients. These facts, together with the problems resulting from the limited options in the treatment and the increased resources imposed by its management, make AKI a focus of growing interest among professionals managing critically ill patients.

